# E2 Proteins of High Risk Human Papillomaviruses Down-Modulate STING and IFN-κ Transcription in Keratinocytes

**DOI:** 10.1371/journal.pone.0091473

**Published:** 2014-03-10

**Authors:** Nuchsupha Sunthamala, Francoise Thierry, Sebastien Teissier, Chamsai Pientong, Bunkerd Kongyingyoes, Thumwadee Tangsiriwatthana, Ussanee Sangkomkamhang, Tipaya Ekalaksananan

**Affiliations:** 1 Department of Microbiology, Faculty of Medicine, Khon Kaen University, Khon Kaen, Thailand; 2 Papillomavirus Regulation and Cancer, Institute of Medical Biology, Agency for Science, Technology and Research (A*STAR), Singapore, Singapore; 3 Department of Pharmacology, Faculty of Medicine, Khon Kaen University, Khon Kaen, Thailand; 4 Department of Obstetrics and Gynecology, Khon Kaen Hospital, Khon Kaen, Thailand; University of Nebraska - Lincoln, United States of America

## Abstract

In the early stages of human papillomavirus (HPV) infection, the viral proteins elicit specific immune responses that can participate to regression of ano-genital lesions. HPV E6 protein for instance can reduce type I interferon (IFN) including IFN-κ that is involved in immune evasion and HPV persistence**.** To evaluate the role of E2 protein in innate immunity in HPV16-associated cervical lesions, genome-wide expression profiling of human primary keratinocytes (HPK) transduced by HPV16 E2 was investigated using microarrays and innate immunity associated genes were specifically analyzed. The analyses showed that the expression of 779 genes was modulated by HPV16E2 and 92 of them were genes associated with innate immunity. Notably IFN-κ and STING were suppressed in HPK expressing the E2 proteins of HPV16 or HPV18 and the trans-activation amino-terminal domain of E2 was involved in the suppressive effect. The relationship between STING, IFN-κ and interferon stimulated genes (ISGs) in HPK was confirmed by gene silencing and real time PCR. The expression of STING and IFN-κ were further determined in clinical specimens by real time PCR. STING and IFN-κ were down-modulated in HPV positive low grade squamous intraepithelial lesions compared with HPV negative controls. This study demonstrates that E2 proteins of high risk HPV reduce STING and IFN-κ transcription and its downstream target genes that might be an immune evasion mechanism involved in HPV persistence and cervical cancer development.

## Introduction

Human papillomavirus (HPV) infection is the most common sexually transmitted infection with approximately 50–80% of sexually active adults acquiring one or more HPV types during their lifetime. The risk of infection is depending on numerous factors including host factors such as the number of sexual partners, age of sexual activity and genetic background as well as viral factors such as the specific viral genotype [1], [2]. HPV genotypes infecting the ano-genital mucosal epithelium are categorized as ‘low-risk’ (LR-HPV) or ‘high-risk’ types (HR-HPV), depending on their association with either benign lesions or lesions that can progress to cancers [3]. Specifically, HR-HPVs are now recognized as the etiological agents of cervical cancer [4]. HPV life cycle is dependent on the differentiation program that keratinocytes undergo within the infected stratified epithelium. Once HPV infects the epithelium through micro-abrasion, the incubation period for production of mature viral particles is from few weeks to years depending on many factors including the infectious dose. HPV infections are very common and most of them remain asymptomatic and are cleared within 6–12 months through an effective host immune response [5]. The cell mediated immune responses to viral early proteins, such as E2 and E6 for instance [6], are probably involved in spontaneous regression of cervical lesions that is accompanied or followed by circulating antibodies or sero-conversion to the major capsid protein L1 [7]. However, approximately 10–20% of infected women have persisting HR-HPV infection and progress to high grade cervical intra-epithelial neoplasia, CIN2/3 and then invasive cancer [8]. Thus, lack of effective immune responses is a potent factor to carcinogenic progression.

The innate immune system has evolved as the first line of host defense against a variety of pathogens. Nucleic acids of pathogens are potent inducers of cellular innate immune defenses after infection [9]–[11]. The initiation of innate immune responses relies on the recognition of pathogen components by germline encoded pattern-recognition receptors (PRRs), including the membrane-bound Toll-like receptors family (TLRs) [12], the retinoic acid-inducible gene I-like receptors family (RLRs) [13], the nucleotide oligomerization domain-like receptors family (NLRs), and cytosolic DNA sensors [14]. Upon recognition, they initiate signal transduction pathways leading to the induction of type I IFN and pro-inflammatory cytokines, which are required for innate immune responses. In this regards, several previous reports indicated that HPV early proteins play major roles in immune evasion of virally induced lesions. For example, E5, E6, and E7 proteins were shown to inhibit delivery of major histocompatibility (MHC) class I molecules, as well as antigen processing and presentation [15]–[17]. E6 and E7 also disrupt the expression and functions of type I IFN [18], [19] via interferon response factors (IRF) type -1 [20] and type-3 [21]. E6 can also bind tyrosine kinase (Tyk) 2 and inhibit its binding to the cytoplasmic portion of the IFN-α receptor 1 therefore inhibiting phosphorylation of Tyk2, as well as signal transducers and activators of transcription STAT-1 and STAT-2, causing the defect in Janus kinase (JAK) and STAT activation, which interferes with IFN-α mediated signaling [22]. Human keratinocytes are the main target of HPV infection in vivo [5]. The E2 protein, which is expressed at the early stage of the HPV life cycle, is a 42-kDa mostly nuclear protein containing three functional domains that are relatively conserved among all papillomaviruses [23], consisting of an amino-terminal trans-activation domain (TAD), a proline-rich hinge region and a carboxy-terminal dimerization DNA binding domain (DBD) [24]. HPV E2 plays essential roles in the viral life cycle by activating viral DNA replication in concert with the E1 viral helicase [25] and regulating viral genome maintenance by its association with the cellular bromo-domain 4 (Brd4) protein [26]. HPV8 E2 protein down-regulates β4-integrin (ITGB4) expression in normal human keratinocytes by displacement of the activator protein 1 (AP-1) cellular transcriptional factor (Jun-B/Fra-1) from the ITGB4 promoter [27], thus mediating transcriptional activation of SF2/ASF [28]. These previous data thus indicate that HPV16 E2 can modulate transcription of both viral and cellular genes. In addition, HPV E2 proteins can interact with many cellular proteins involved in transcriptional regulation including SMCX, EP400, BRD4, TopBP1, hSNF5 (SW1/SNF component), TAF1, TBP1, TFIID and TFIIB, topoisomerase I, BRCA1, p/CAP, CBP, PARP (poly(ADP-ribose) polymerase 1), p300, Sp1, AMF-1/Gps2, hNAP-1, BRM, and Mdm2 (reviewed in [29]). Since HPV E2 protein is involved in a large variety of cellular functions, we decided to investigate its putative role in the context of immune evasion. Genome-wide expression profiling of undifferentiated human primary keratinocytes (HPK) transduced with HPV16E2 expression vectors was investigated for innate immune associated genes. The results showed that stimulator of interferon genes (STING) and IFN-κ were specifically down-modulated in human keratinocytes transiently expressing HPV16E2. STING silencing revealed a link between STING and IFN-κ in these cells and IFN-κ silencing lead to transcriptional inhibition of downstream interferon stimulated target genes (ISGs). Abrogation of IFN-κ expression may represent an early and central key event in the progression of HPV-infected lesions and ultimately in the development of cervical cancer.

## Materials and Methods

### Clinical Specimens

Fresh cervical tissue biopsies were obtained from 148 patients recruited at the Gynecology Department, Srinagarind Hospital, Faculty of Medicine, Khon Kaen University, Thailand. The Ethics Committee of Khon Kaen University has approved this study (no. HE541377) and all subjects gave written informed consents for participation. HPV infection status of the cervical tissue biopsies was determined by PCR and HPV reverse line blot hybridization.

### Cell Culture

Human primary keratinocytes were used in this study including human primary neonatal foreskin keratinocytes (HPK) (Lonza, Basel, Switzerland), human primary adult foreskin keratinocytes single donor NUH49 and TB3 (HPEKas.05, CELLnTEC, Bern, Switzerland). The keratinocytes were cultured in supplemented CnT-57 medium (CELLnTEC, Bern, Switzerland). All cells used were tested and found free of mycoplasma.

### Transduction

Recombinant adenoviruses containing GFP, GFP-HPV16 E2, GFP-HPV18 E2, and 2 truncated forms of HPV18 E2, GFP-HPV18 E2TAD (containing the amino-terminal transactivation domain of HPV18 E2), and GFP-HPV18 E2DBD (containing the DNA binding domain of HPV18 E2) were used as previously described [30], [31]. Keratinocytes were allowed to attach on plate surface for 24 h and were then transduced with recombinant adenoviruses at a multiplicity of infection (m.o.i.) of 50 [32]. These conditions of infection allowed ∼90% positively transduced cells expressing GFP that were collected by scraping for RNA extraction after 48 h.

### Gene Silencing in HPK

Gene silencing of STING and IFN-κ was performed using siRNA prepared against STING, IFN-κ, and a siControl (Dharmacon Thermo Fisher Scientific, New Hampshire, USA). The siRNA were transfected to HPK cells using Lipofectamine RNAiMax (Invitrogen, Carlsbad, CA, USA). Briefly, 80 nmol of siRNA was added to 500 µl Opti-MEM (Gibco Life Technologies, Invitrogen, Carlsbad, CA, USA) in 6-well plates. Lipofectamine RNAiMax was added, gently mixed and incubated at room temperature for 15 min. 2×10^4^ cells of HPK were re-suspended in 2.5 ml of supplemented CnT-57 medium and were gently placed to the well containing the siRNA mixture. The cells were incubated for 48 h and then harvested for RNA extraction.

### Total RNA Isolation and Quantitative RT-PCR

DNA and RNA were extracted from fresh cervical tissue biopsies by AllPrep DNA/RNA Mini Kit (QIAGEN, Singapore). The extracted RNA was reverse transcribed using reverse transcriptase (Invitrogen, Carlsbad, CA, USA) and random hexamer (Promega, WI, USA). PCR reactions were performed with gene-specific primers for STING, IFN-κ, and house-keeping gene GAPDH (Sigma-Aldrich, MO, USA).

Total RNA from HPK, TB3, and NUH49, that were transduced with recombinant adenoviruses GFP, GFP-HPV16 E2, GFP-HPV18 E2, GFP-HPV18 E2TAD, and GFP-HPV18 E2DBD, were isolated using RNeasy mini kit (QIAGEN, Singapore) followed by RNeasy mini protocol. Total RNA (0.2 µg) was reverse transcribed using reverse transcriptase (Invitrogen, Carlsbad, CA, USA) and random hexamer (Promega, WI, USA). Duplicate PCR reactions were performed with gene-specific primers for STING, IFN-κ, IFIT1, IFIT3, OAS1, OAS2, OAS3, MX1, MX2, PKR, GFP, and GAPDH (Sigma-Aldrich, MO, USA). Real-time PCR was performed using SYBR Green PCR Master Mix (Applied Biosystems, Invitrogen, Carlsbad, CA, USA). Threshold cycle numbers (CT) were determined with 7900HT Fast Real-time PCR System (Applied Biosystems, Invitrogen, Carlsbad, CA, USA) and the relative quantities of mRNA per sample were calculated using the ΔΔCT method using GAPDH as the calibrator gene. The relative levels of mRNA were determined by setting the mRNA expression level of the lowest expression control to 1.

### cRNA Synthesis and Microarray Hybridization

Triplicate of GFP and GFP-HPV16 E2 transduced HPK cells were harvested after 48 h of transduction. Total RNA were isolated and analyzed on an RNA 6000 Nano Lab-on-a-Chip in the 2100 Bioanalyzer (Agilent Technology, ON, CA), showing RIN score above 9.4. 200 ng of RNA were labeled using Illumina TotalPrep-96 RNA Amplification kit (Ambion Life Technologies, Invitrogen, Carlsbad, CA, USA) as per amplification protocol. 750 ng of cRNA generated from amplification and labeling were hybridized into one Human HT-12 v4.0 BeadChip. The BeadChip was incubated at 58°C, with rotation speed 5 for 18 h for hybridization. The BeadChip was washed and stained as per the Illumina protocol and scanned on the iScan (Illumina, CA, USA). The data files were quantified in GenomeStudio Version 2011.1 (Illumina, CA, USA). All samples passed Illumina’s sample dependent and independent QC metrics.

### Analysis of Differential Gene Transcription

Gene expression data were imported into Partek Genomics Suite 6.5 (Partek, St Louis, Mo). Raw data were preprocessed, in steps of background correction, normalization, and summarization using robust multi-array average analyses, and the expression data were transformed to log2. Principal-component analysis (PCA) was performed to identify outliers of sample group. Differential expression analysis for the samples was performed using one way analysis of variance (ANOVA). Gene lists were created using a cut-off of *P*≤0.05, 1.5-fold change. Hierarchical clustering was performed using the Gene Expression module. Gene Ontology (GO), an established gene database, was used to define biological categories and functions. Network analysis was performed using core analysis of Ingenuity Pathways Analysis (IPA) to construct the network from microarray gene list. Signaling pathways were analyzed using Kyoto Encyclopedia of Genes and Genomes (KEGG) database to define the top of signaling pathways that were altered by HPV16 E2. The microarray data was deposited at Gene Expression Omnibus with accession no GSE54008.

### Statistical Analysis

All real-time PCR data were expressed as mean ± SD. Statistical analyses were performed with SPSS using ANOVA. The groups in each experiment were three or more. Following ANOVA, a post hoc test was performed using the Least Significant Difference test (LSD) and Tukey’s Honestly Significant difference test (Tukey’s HSD). If *P*≤0.05, results are considered statistically significant.

## Results

### HPV GFP-E2 Fusion Proteins Expression and Function From Recombinant Adenoviruses -transduced Cells

**Figure 1:**
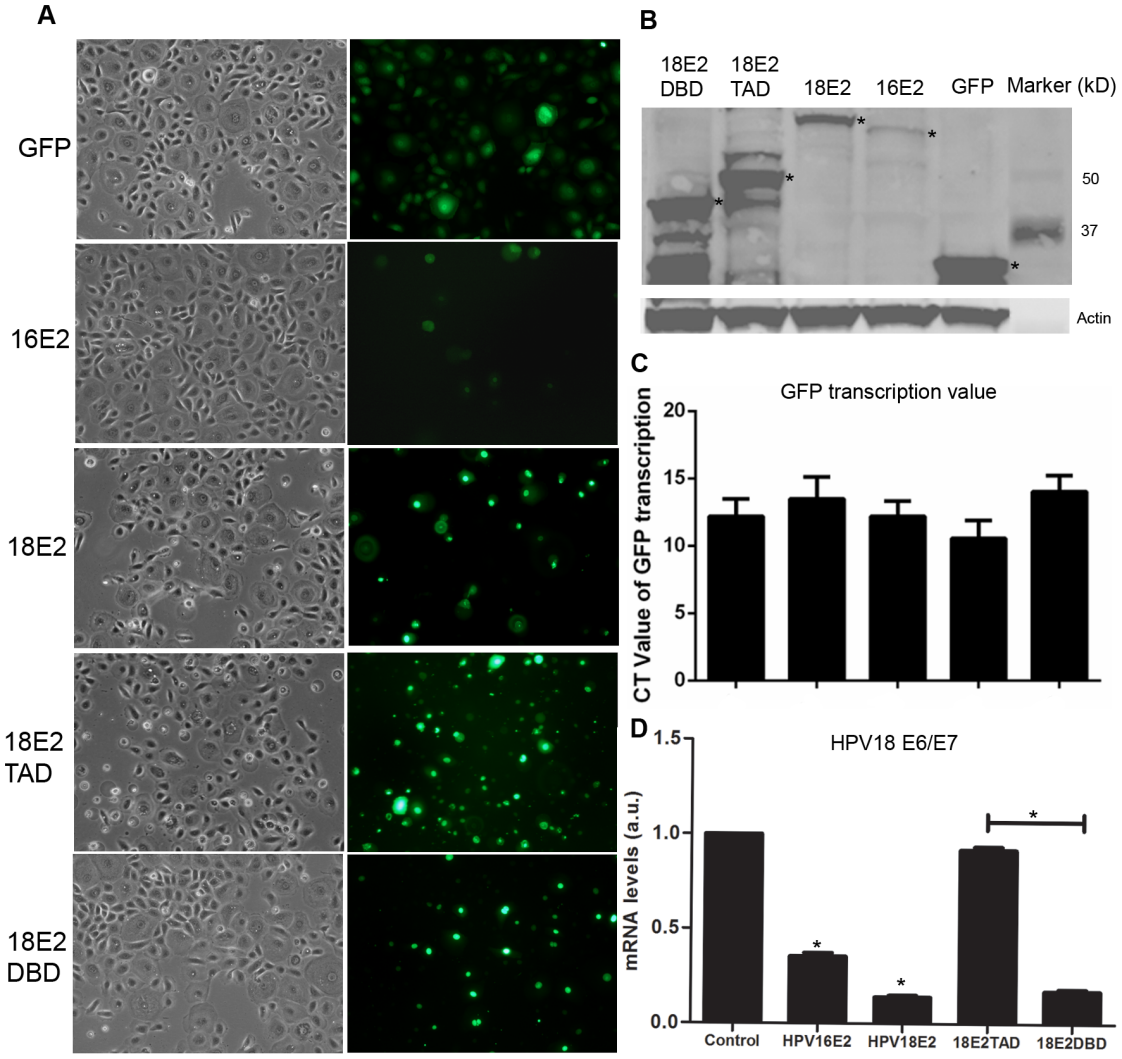
HPV E2s expression in recombinant adenovirus HPV E2-transduced human primary keratinocytes. (A) GFP signals of recombinant adenoviruses GFP, GFP-HPV16 E2, GFP-HPV18 E2, GFP-HPV18 E2TAD, and GFP-HPV18 E2DBD under fluorescence microscope on live cells (10X). (B) GFP protein expression was detected by western blotting analysis. GFP, GFP-HPV16 E2, GFP-HPV18 E2, GFP-HPV18 E2TAD, and GFP-HPV18 E2DBD showed the expected protein sizes of ∼32 kD, 62 kD, 64 kD, 50 kD, and 45 kD, respectively and are indicated by asterisks. The β-actin protein was used as loading control. (C) The mRNA expression levels of recombinant adenoviruses GFP, GFP-HPV16 E2, GFP-HPV18 E2, GFP-HPV18 E2TAD, and GFP-HPV18 E2DBD are shown as CT values (mean±SD). (D) The suppressive effect of HPV E2 on HPV18 E6/E7 transcription in HeLa cells is shown as relative transcripts levels compared to cells not expressing E2 using real-time PCR. (*P≤0.05).

The recombinant adenoviruses containing GFP-HPV E2s were used as described in previous studies [30]–[32] to transduced several cell types. Since HPV target cells in vivo are basal epithelium, undifferentiated human keratinocytes (HPK) were used to study genes modification by HPV16 E2 in vitro. Recombinant adenoviruses containing GFP, GFP-HPV16 E2, GFP-HPV18 E2, GFP-HPV18 E2TAD, and GFP-HPV18 E2DBD were transduced to HPK at various m.o.i. (200, 100, 50, and 25, respectively, data not shown). The m.o.i. 50 was defined as the suitable m.o.i. that gave rise to ∼100% GFP positive cells and quantifiable E2 mRNA expression in human keratinocytes. Intensity of the GFP signals under a fluorescence microscope were comparable in cells transduced with the various recombinant adenoviruses although GFP-HPV16 E2 exhibited a weaker GFP signal than other GFP fusion proteins (Fig. 1A). Partial cytoplasmic localization of the GFP fusion proteins was found in the cells transduced with GFP-HPV16E2, GFP-HPV18E2, and GFP-HPV18E2TAD whereas the GFP-HPV18E2DBD was mostly found in nucleus. Western blot analysis revealed a specific low expression of GFP-HPV16 E2 compared with other fusion proteins (Fig. 1B) whereas the mRNA levels were comparable in all transduced HPK (Fig. 1C).

Overexpression of wild type HPV E2 proteins can induce growth arrest of HeLa cells by repressing E6/E7 transcription via repression of the endogenous integrated HPV18 *E6/E7* promoter [33], [34]. Several papillomaviruses such as the carcinogenic HPV31 express, in addition to full length E2, an E8ˆE2C fusion protein, which is required for repression of replication and transcription [35], [36]. Thus, the GFP-tagged DBD domain of HPV E2 protein alone exhibits a repressor function on HPV E6/E7 gene promoter. We evaluated the transcriptional repression activity of the GFP-tagged HR-HPV E2 by transducing the various isoforms into HeLa cells and determining the HPV18 E6/E7 mRNA levels by real-time PCR. These experiments indicated that both full-length GFP-HPV16E2 and HPV18E2 showed comparable repressive activity on endogenous HPV18 E6/E7 gene expression in HeLa cells. Analysis of the deleted proteins revealed that HPV18E2DBD is also a potent repressor of HPV18 E6/E7 gene expression but not the TAD domain (Fig. 1D). These results thus show that the GFP-HPV E2 fusion proteins exhibit similar activities as reported earlier for the untagged HPV E2s.

### Genome-wide Profiling of HPV16 E2-transduced HPK

**Figure 2:**
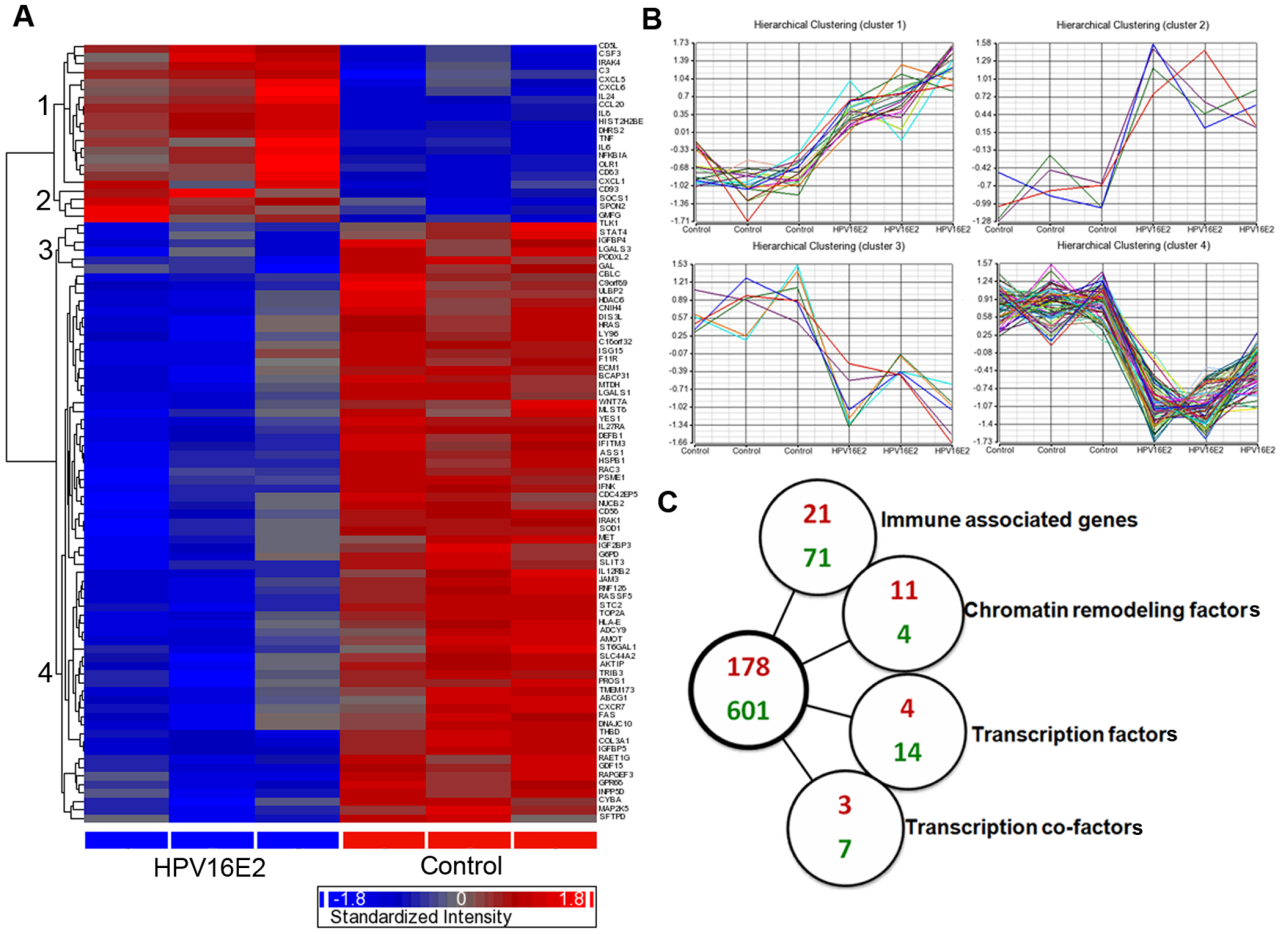
Genome-wide profiling in HPK transduced by HPV16 E2. Microarray was performed 48-off of *P*<0.05, 1.5-fold change. (A) Gene Ontology (GO) was used to define biological categories and functions. (B) Hierarchical clustering was constructed from immune associated genes as defined by GO. Cluster 1 and 2 were up-regulated cluster genes and cluster 3 and 4 were down-regulated cluster genes. (C) Signature genes altered in HPK expressing HPV16 E2, immune associated genes were analyzed by GO. Chromatin remodeling factors, transcription factors, and transcription co-factors were analysed by Animal Transcription Factor Database (AnimalTFDB), red numbers represent up-regulated genes; Green numbers represent down-regulated genes.

To identify cellular gene expression profile modified by HPV16 E2, the expression profiles of GFP-HPV16 E2 HPK transduced cells were compared with those of GFP HPK transduced cells as a negative control. Data analysis indicated that HPV16 E2 up-regulated 178 genes and down-regulated 601 genes. Gene Ontology analysis further revealed that 21 up-regulated genes and 71 down-regulated genes are involved in immune responses. Hierarchical clustering was constructed using the 92 immune associated genes modulated (Fig. 2A and B). The complete list of genes (fold-change ±1.5 compare to GFP control) is shown in supplement Table S1. These data demonstrate that HPV16 E2 is a potent modulator of keratinocyte gene expression, including immune associated genes. Altered cellular signaling pathways in HPK expressing HPV16 E2 based on KEGG database are shown in Table 1. The top 10 canonical pathways include metabolic pathway, cytokine-cytokine receptor interaction, pathway in cancer, viral carcinogenesis, protein processing in endoplasmic reticulum, PI3K-AKT signaling pathway, MAPK signaling pathway, leukocyte trans endothelial migration, chemokine signaling, and focal adhesion. The top 10 canonical pathways of up-regulated and down-regulated genes are shown in Table 2.

**Table 1:** Top 10 canonical pathways modulated in HPK expressing HPV16 E2 protein.

Signaling pathways	No. of altered gene
Metabolic pathway	50
Cytokine-cytokine receptor interaction	17
Pathway in cancer	15
Viral carcinogenesis	12
Protein processing in endoplasmic reticulum	11
PI3K-AKT signaling pathway	11
MAPK signaling pathway	9
Leukocyte transendothelial migration	9
Chemokine signaling	9
Focal adhesion	9

**Table 2:** Top 10 modulated pathways in HPK expressing HPV16 E2 protein.

Signaling pathways	No. of altered gene
**Pathway involving up-regulated genes**	
Cytokine-cytokine receptor interaction	10
Viral carcinogenesis	8
Chemokine signaling pathway	6
NOD-like receptor signaling pathway	5
Toll-like receptor signaling pathway	5
Transcriptional misregulation in cancer	5
NF-κ B signaling pathway	4
Jak-STAT signaling pathway	4
Pathway in cancer	4
Apoptosis	4
**Pathway involving down-regulated genes**	
Metabolic pathway	48
Pathway in cancer	11
Protein processing in endoplasmic reticulum	10
Leukocyte transendothelial migration	9
PI3K-AKT signaling pathway	9
MAPK signaling pathway	8
Cytokine-cytokine receptor signaling pathway	7
Wnt signaling pathway	6
Oxidative phosphorylation	6
Natural killer cell mediated cytotoxicity	6

To identify the transcriptional regulation of cellular genes involved in the control of basal transcription by HPV16 E2, the HPV16 E2 regulated genes were analyzed based on Animal Transcription Factor Database (AnimalTFDB) in 4 main functions, chromatin remodeling factors, transcription factors, transcription co-factors, and transcription elongation factors (Fig. 2C). The transcription networks modulated by E2 are depicted as up-regulated genes and down-regulated genes (Table 3).

**Table 3:** Transcription network- associated genes modulated by HPV16E2 in HPK.

Gene function	Gene no.	Gene name
**Up-regulated genes**		
Chromatin remodeling	11	HIST1H4H, HIST1H2BG, HIST1H3D, HIST1H2AC, HIST1H2AE, HIST1H2BD, HIST1H2BC, HIST2H2BE, HIST2H2AA3, HIST2H2AA4, HIST2H2AC
Transcription factors	4	ATF3, GATA4, FOXS1, ZNF24
Transcription co-factors	3	PPP1R1A, NFKBIA, NCOA2
Transcription elongation factor	1	TCEB1P3
**Down-regulated genes**		
Chromatin remodeling	4	ITGB3BP, RBBP7, HDAC6, SMARCA2
Transcription factors	14	NFIB, NFIX, NHLH2, HES2, MNT, ZFP30, ZNF579, GTF3A, ZNF473, ETV2, STAT4, NR2F2, TSC22D3, IRX1
Transcription co-factors	7	CRSP9, RAC3, TGFB1I1, LMO4, BASP1, PEX14, TRIB3

The first immune network analysis was constructed using 90 signature genes from the literature and high-throughput screening in Core Analysis of Ingenuity Pathways Analysis (IPA). It was merged from 5 networks based on main functions of immune responses including immune cell trafficking, inflammatory response, cell-mediated immune response, and humoral immune response. This analysis demonstrated the connectivity of genes in MAPK, NF-κB pathways, as well as the link of MHC class I molecule, complement pathway, cytokines, and chemokines (supplement Fig. S1). The second network was merged from 72 down-modulated genes based on cell-mediated immune response and cell-to-cell signaling and interaction (supplement Fig. S2). STAT4 stands as a central factor that links several genes such as type I IFN and ISGs, playing roles in the immune response to viral infections. Notably, IFN-κ, a member of the type I IFN family, was down-regulated by HPV16 E2 as well as TMEM173 (STING) which has previously been shown to have a link with type I IFN although not specifically with IFN-κ. We therefore decided to determine whether these two genes could be linked in human keratinocytes for the immune response to viral infection.

### STING and IFN-κ were Down-modulated by Full-length E2 and its Amino-terminal Domain

**Figure 3:**
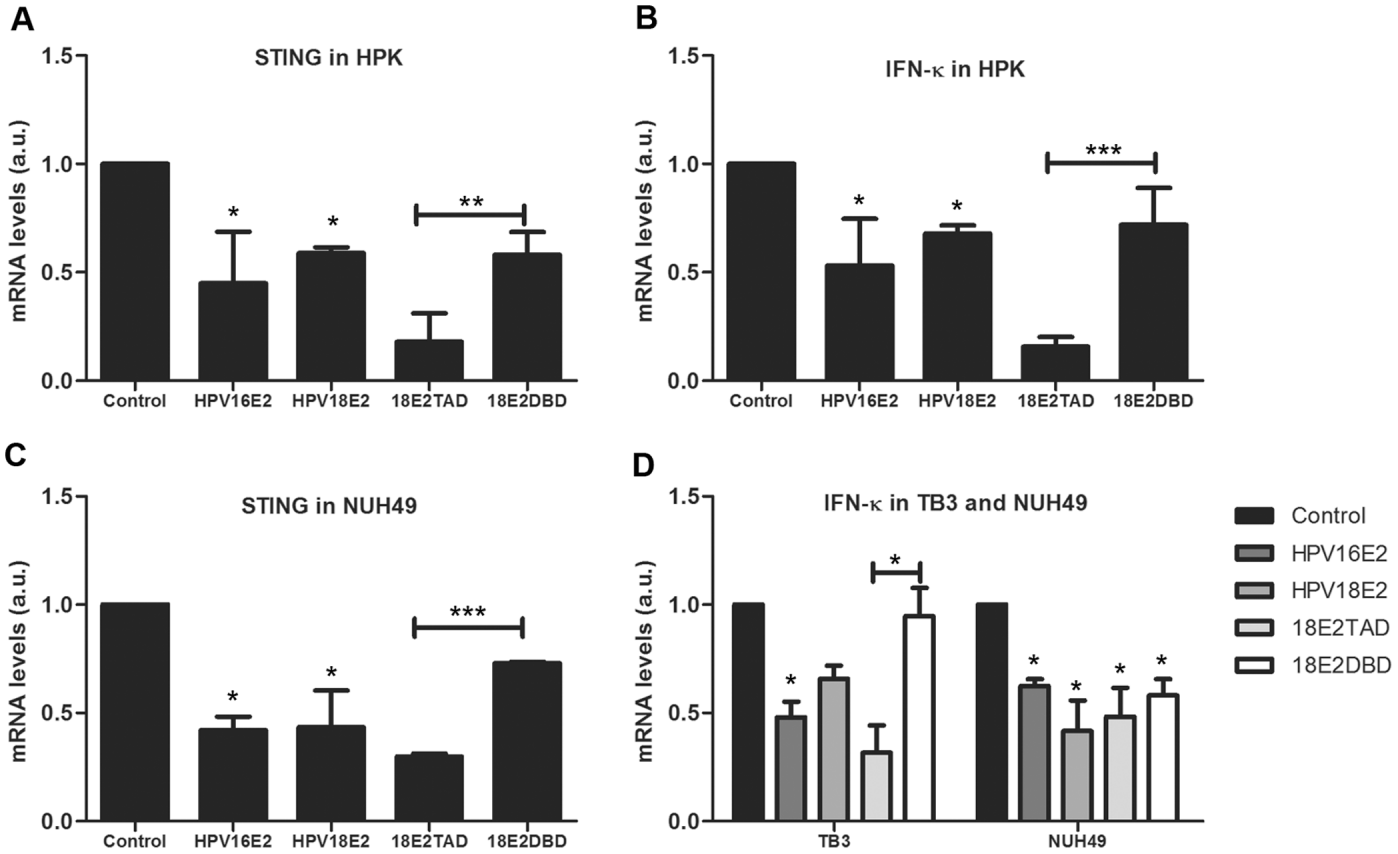
STING and IFN-κ transcripts were down-regulated by HPV E2s. (A) STING transcripts level analyzed by RT-PCR was significantly down-regulated by HPV16 E2, HPV18 E2 and the HPV18 E2 TAD in HPK and B) IFN-κ transcription level was significantly down-regulated by HPV16 E2, HPV18 E2 and HPV18 TAD in HPK. C) STING transcripts level was modulated by E2 in NUH49 as shown in (A). D) IFN-κ transcripts level was modulated by E2 in ITB3 and NUH49 as in (B). (A–D) HPV18 E2TAD significantly down-regulated STING and IFN-κ genes transcription when compared to HPV18 E2DBD. (*P≤0.05, **P≤0.01, ***P≤0.001).

To verify the modulation of STING and IFN-κ transcription in human keratinocytes, recombinant adenoviruses containing GFP, GFP-HPV16E2, GFP-HPV18E2 were transduced into 3 different primary human keratinocytes cells HPK, TB3, and NUH49. Real-time PCR analyses demonstrated that E2 proteins of HPV16 and 18 potently down-modulated STING mRNA levels in HPK and NUH49 keratinocytes (Fig. 3A and 3B). IFN-κ was also down-regulated by HPV16 and HPV18 E2s in HPK, TB3, and NUH49 when compared to adenovirus GFP control transduced keratinocytes (Fig. 3C and 3D).

To evaluate which domain of HPV E2 is responsible for STING and IFN-κ regulation, recombinant adenoviruses containing GFP, GFP-HPV18 E2TAD, GFP-HPV18 E2DBD were transduced to HPK, TB3, and NUH49. STING transcripts levels were down-regulated in HPK and NUH49 expressing HPV18 E2TAD when compared to HPV18 E2DBD expressing cells (Fig. 3A and 3B). IFN-κ transcripts levels were also down-modulated by HPV18 E2TAD in HPK, TB3, and NUH49 (Fig. 3C and 3D), indicating that the amino-terminal domain of E2 (TAD) plays an essential role in STING and IFN-κ gene regulation and not the DNA binding domain.

### IFN-κ is Regulated by STING in Human Keratinocytes

**Figure 4:**
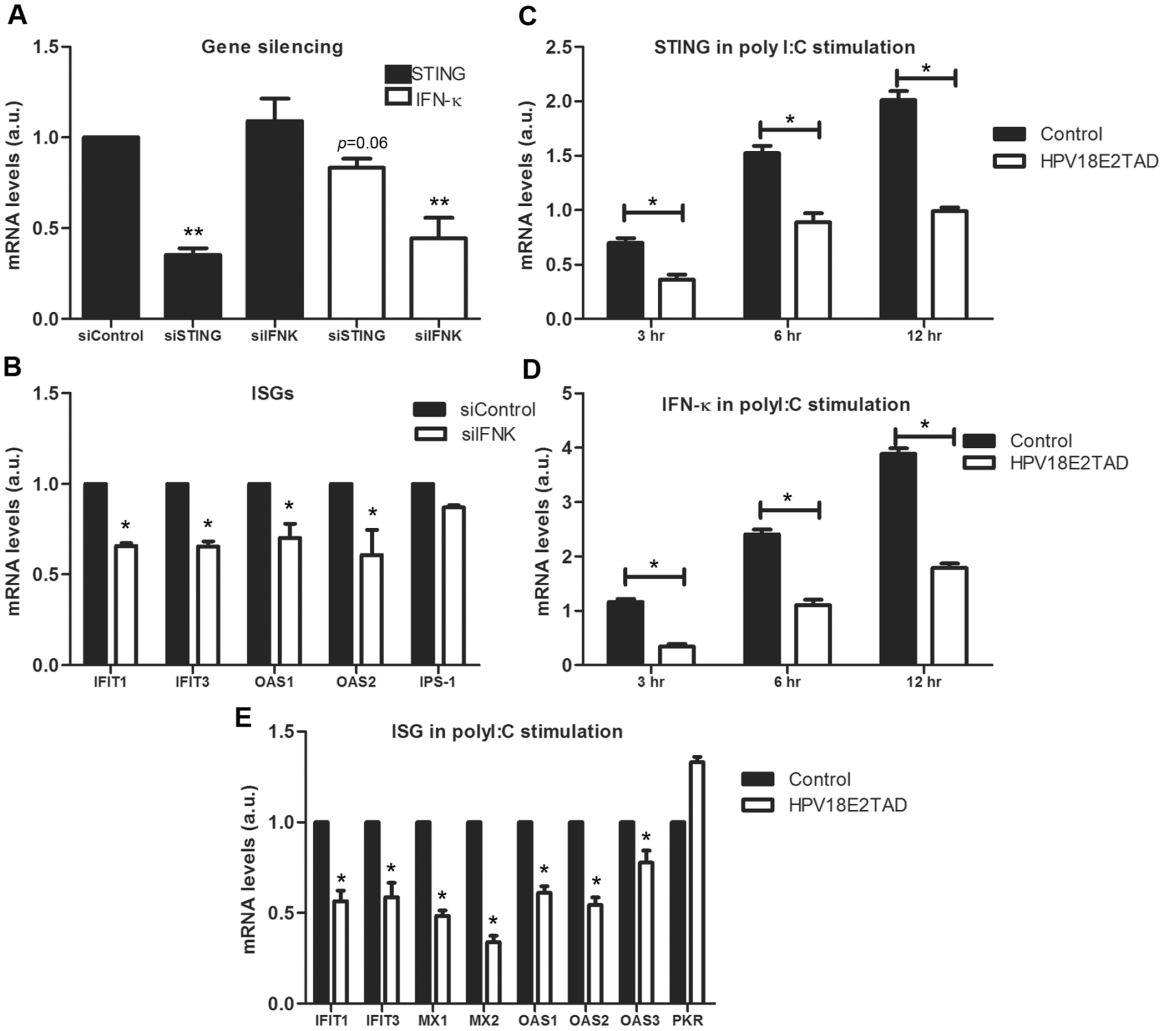
Transcriptional reduction of IFN-κ in STING-silenced HPK and poly I:C stimulated HPK. (A) STING and IFN-κ transcription level in HPK cells after transfection with siSTING and siIFNK for 48 h. IFN-κ transcription level in STING-knockdown HPK was reduced by ∼20% (*P* = 0.06). STING transcription level in IFN-κ-knockdown HPK was not changed. (B) ISGs transcription levels were down-modulated in IFN-κ-knockdown HPK. (C) TAD of HPV18 E2 abrogated STING transcription in Poly I:C induced HPK cells. Cells transduced with GFP as control and GFP-E218TAD were stimulated with poly I:C (10 µg/ml) for 3, 6, and 12 h. STING transcription level was measured at each time point. (D) IFN-κ transcription level was abrogated by HPV18 E2TAD when examined at each time point of IFN-κ induction by poly I:C (E) ISGs were determined after stimulation of HPK expressing HPV18 E2TAD with poly I:C (10 µg/ml) for 3 h. (*P≤0.05, **P≤0.01, ***P≤0.001).

STING can activate both NF-κB and IRF3 transcription pathways to induce expression of members of the type I IFN (IFN-α and IFN-β) and thereby exert a potent antiviral effect when expressed [37]. However, the association of STING and IFN-κ in keratinocytes is not known. To determine whether a functional link exists between these two genes, gene silencing experiments were performed in human keratinocytes to knockdown STING or IFN-κ transcription upon siRNA tranfection reaching a ∼70% efficiency (Fig. 4A). IFN-κ transcription was down-modulated to around 20% by silencing of STING (*P* = 0.06) whereas silencing of IFN-κ did not affect STING transcriptional level (Fig. 4A), suggesting that STING might act as an upstream effector of IFN-κ gene expression in human keratinocytes. Silencing of IFN-κ reduced ISGs transcription such as IFIT1, IFIT3, OAS1, and OAS2 transcription levels as expected (Fig. 4B).

### HPV18 E2TAD Abrogated STING and IFN-κ Induction and Suppressed ISGs Transcription

To further evaluate the role of the E2 amino-terminal domain (TAD), HPK cells were transduced by recombinant adenoviruses GFP, and GFP-HPV18 E2TAD for 48 hrs after which transduced keratinocytes were stimulated with poly I:C (10 µg/ml) for an additional 3, 6, and 12 hrs. As measured by RT-PCR, STING and IFN-κ transcription levels were significantly reduced in HPV18 E2TAD infected HPK cells at each time point of poly I:C activation compared to GFP-control cells (Fig. 4C and 4D, respectively). HPV18 E2TAD also down-modulated several ISG gene expression including, IFIT3, IFIT1, MX1, MX2, OAS1, OAS2, OAS3, whereas in contrast PKR was up-regulated (Fig. 4E). These data clearly demonstrated that the E2TAD alone could efficiently down modulate the stimulated IFN pathway in transduced human keratinocytes in vitro and that E2TAD-mediated effect was even more potent than siRNA knock-down of IFN-κ.

### STING and IFN-κ were Down-modulated in HPV Positive Clinical Specimens

**Figure 5:**
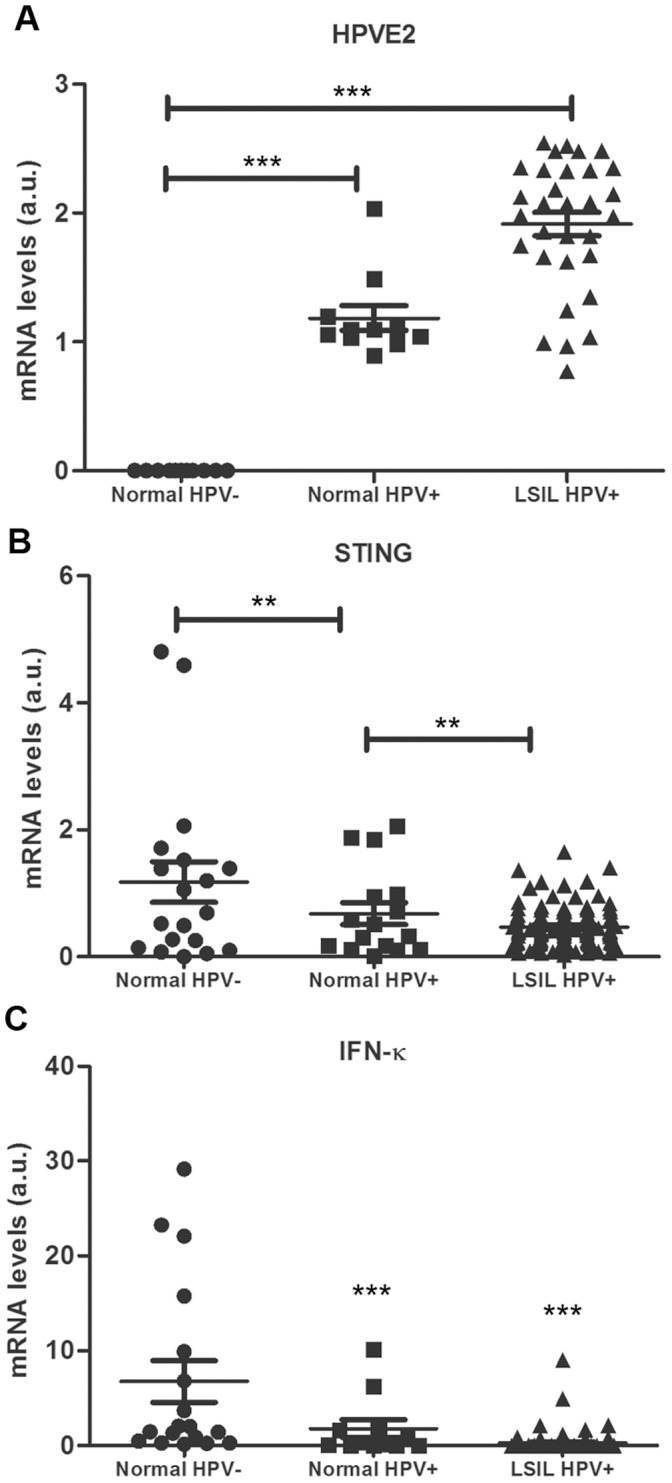
STING and IFN-κ down-regulation in HPV positive clinical specimens. (A) HPV E2s transcription level was significantly increased in HPV positive normal and LSIL when compared to HPV negative normal cases (*P*<0.05). (B) STING transcription level was significantly decreased in LSIL with HPV positive and HPV E2 positive cases (*P*<0.05). Normal cases with HPV positive also showed low STING transcription level when compared to normal HPV negative cases (*P*<0.05). (C) IFN-κ transcription level was significantly down-regulated in normal HPV positive cases and HPV positive LSIL cases when compared to normal HPV negative cases (*P*<0.05). (*P≤0.05, **P≤0.01, ***P≤0.001).

To determine the effect of HPV infection on STING and IFN-κ expression in vivo in patients lesions, 148 clinical specimens were collected and their HPV infectious status and grade were examined by histology, HPV detection, and HPV genotyping. Based on histological analyses, clinical samples were graded into cervicitis or no intra-epithelial lesion (inflammation) and low grade squamous intra-epithelial lesion (LSIL). Samples of both stages were examined for HPV DNA and HPV E2 transcription. HPV E2 transcriptional levels were significantly increased in HPV positive normal and LSIL when compared to HPV negative normal cases (Fig. 5A). STING and IFN-κ mRNAs expression levels were also examined. STING transcriptional level was significantly lower in HPV positive LSIL cases compared with HPV negative or inflammation group (Fig. 5B). IFN-κ transcription level was also significantly down-regulated in normal and HPV positive LSIL cases compared to the HPV negative inflammation group (Fig. 5C).

## Discussion

Our study revealed an unexpectedly large modulation of cellular genes by HPV16 E2 in human keratinocytes in the absence of any other viral gene expression. Several cell signaling pathways were modulated (Table 1 and 2) including immune and non-immune pathways, which showed some overlap with the gene profiling of E2 expression in a HPV negative cervical cancer cell line, C33A [38]. In the present report, the transcriptional networks were analyzed based on AnimalTFDB and the gene profiling data indicated that HPV16 E2 can modulate the cellular transcriptional apparatus, including many transcription factors and co-factors (Table 3). In addition, several histones were up-regulated while the repressor histone deacetylase HDAC6 was down-modulated. These transcriptional modulations might affect downstream genes and lead to epigenetic modification of the host cell genome.

The cytosolic DNA recognition leads to activation of TANK-binding kinase 1 (TBK1) and IRF-3 and production of type I IFNs. TBK1 associates with DDX3, a DEAD box upstream RNA helicase, which regulates IFN-β transcription via IRF-3. TBK1 interacts with the exocyst protein Sec5 in a complex that includes the recently identified endoplasmic reticulum adaptor STING [37], [39]. STING interacts with TBK1 and IRF-3 leading to the phosphorylation of IRF-3 and production of type I IFN [40]. IFN-κ is a member of type I IFN family that is expressed in human keratinocytes and also can be detected in dendritic cells [41]. IFN-κ is reduced in HPV-infected keratinocytes [42] via *de novo* methylation on a CpG island near the transcriptional start site induced by HPV16 E6 [43], [44]. The reduction of IFN-κ results in the decrease of several ISGs and leads to impaired host cell antiviral responses. STING contributes to stimulating IFN-β as well as IFN-α, but the correlation between STING and IFN-κ was not demonstrated. The present report shows that the E2TAD can reduce the poly I:C induced activation of several ISGs. The well characterized IFN-induced pathways are the anti-viral pathways, the PKR, the OAS, and the MX pathways [45]–[47]. IFN-κ activates the ISRE pathway and signal transduction through the type I IFN receptor [41] which leads to induction of ISGs. Interestingly, our results indicate that HPV16 E2 mediated down-regulation of STING in HPK might lead to concomitant reduction in IFN-κ expression. The reduction of IFN-κ by HPV18 E2 TAD leads to the down modulation of the ISGs genes tested in this report, namely IFIT3, IFIT1, MX1, MX2, OAS1, OAS2, and OAS3. Transcriptional expression of the selected ISGs in polyI:C stimulated HPK cells was more efficiently down modulated by E2 than in IFN-κ knockdown cells. This might be explained by a better efficiency of polyI:C activation together with E2TAD expression. The E2 TAD has been shown to interact with numerous cellular regulatory proteins (reviewed in [48]), as well as the full length E2 protein [29]. E2TAD also interacts with the E1 helicase that activates viral DNA replication [49], [50] and with Brd4 involved in episomal segregation during mitosis [51]. Other E2 functions, such as stability of the protein [52] as well as induction of apoptosis, involve this domain [30]. Induction of apoptosis is caused by accumulation of E2 in the cytoplasm and involves caspase 8 activation [31]. An high-through put yeast two hybrid analysis of several HPV E2 proteins revealed the interaction of HPV E2s with numerous cellular proteins that can alter the cellular functions including transcription regulation, regulation of apoptosis, RNA processing, ubiquitination and intracellular trafficking [53], [54]. Accordingly, the suppression of STING and IFN-κ gene transcription in human keratinocytes by E2TAD might be mediated by protein interaction of HPV18E2TAD with several specific cellular proteins. These interactions might be direct or indirect either in the nucleus or cytoplasm of transduced cells that ultimately would lead to transcriptional repression. Moreover, our microarray data contain several cellular transcription factors which are regulated by HPV16E2, that could be regulators of STING and IFN-κ gene expression.

IFN-κ was strongly down-regulated in normal and LSIL HPV positive cases compared to HPV negative control tissues. This phenomenon might be induced by several HPV early proteins including the viral oncoproteins E6 and E7, but in the early stage of HPV infection, E2 is highly expressed and might therefore also play a major role to abrogate IFN-κ transcription [29], [43], [44]. The mechanisms of the suppression of IFN-κ production by HPV16 E2 should be clarified in future work. However this is the first report focusing on a putative role of HPV E2 in mechanisms of immune evasion. This novel function of HPV E2 in regulating immune response associated genes thereby implicates this viral protein in the persistence of viral infection ultimately leading to cervical cancer.

## Supporting Information

Figure S1 **The immune response-associated network.** The network was merged based on main functions of immune responses, immune cell trafficking, inflammatory response, cell-mediated immune response, and humoral immune response, which involved 90 gene products. This network demonstrated the connectivity of genes in MAPK, NF-κB pathways, as well as the link between MHC class I molecule, complement pathway, cytokines, and chemokines. Molecules are represented as nodes, and the biological relationship between two nodes is represented as an edge (line). Green, down-regulated genes; red, up-regulated genes; gray, not differentially expressed; solid line, direct interaction; dashed line, indirect interaction; yellow line, merged interaction.
(TIF)

Figure S2 **The cell-mediated immune responses-associated network.** The network was merged from 72 E2 mediated down-regulated cellular genes based on cell-mediated immune response and cell-to-cell signaling and interaction. This network showed a connection between IFN-κ and STING (TMEM173) with type I IFN being central. Molecules are represented as nodes, and the biological relationship between two nodes is represented as an edge (line). Green, down-regulated genes; gray, not differentially expressed; solid line, direct interaction; dashed line, indirect interaction; yellow line, merged interaction.
(TIF)

Table S1 **Immune responses associated genes modulated by HPV16 E2 in HPK.** Gene Ontology analysis indicated that 21 up-regulated genes and 71 down-regulated genes by HPV16 E2 are involved in immune responses.
(DOC)
