# Supplementary figures and images for: E2 Proteins of High Risk Human Papillomaviruses Down-Modulate STING and IFN-κ Transcription in Keratinocytes
Source: PLoS One. 2014 Mar 10;9(3):e91473. doi: 10.1371/journal.pone.0091473 (PMC3948877; doi:10.1371/journal.pone.0091473)

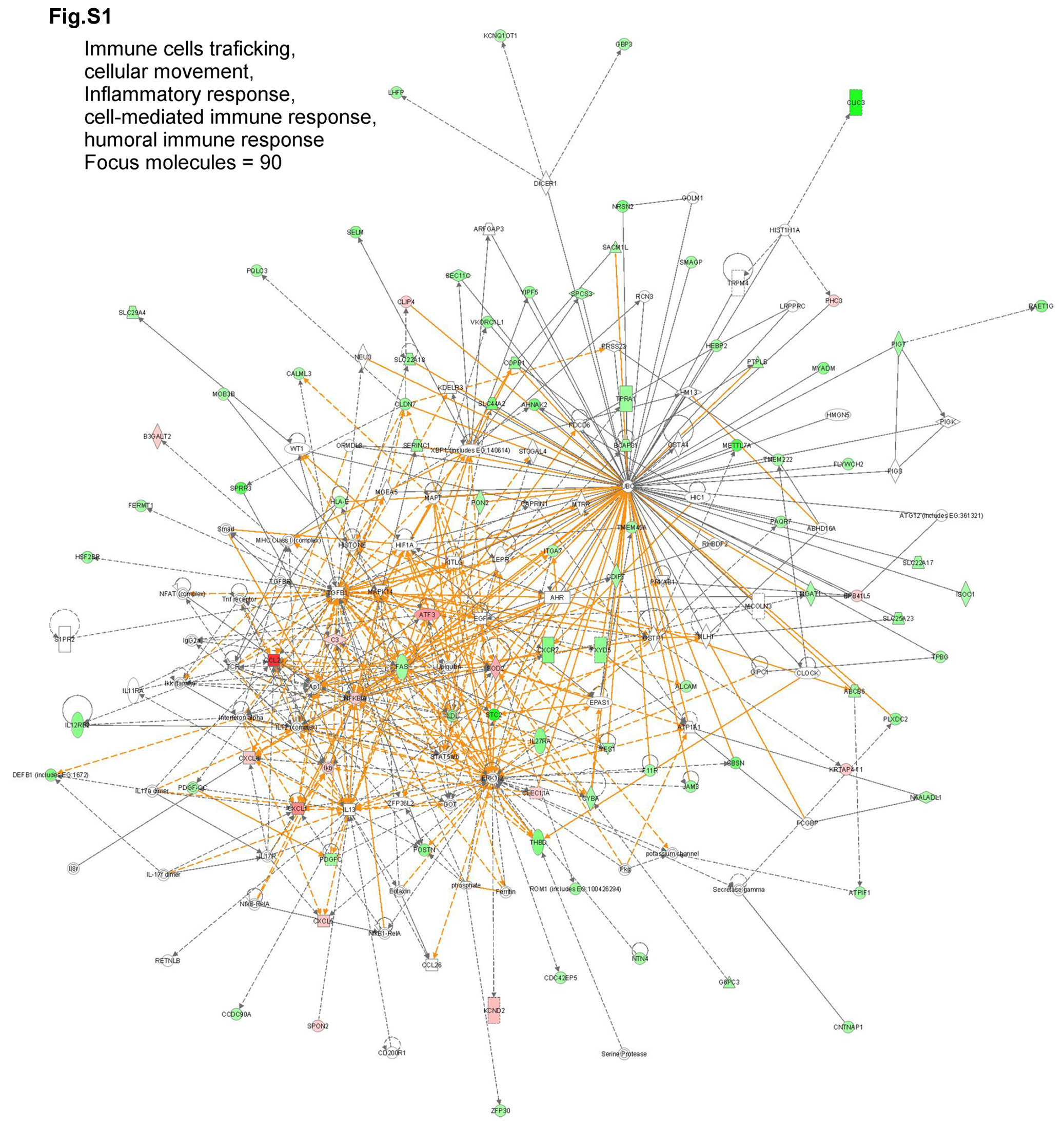

Supplement: Figure S1 — The immune response-associated network. The network was merged based on main functions of immune responses, immune cell trafficking, inflammatory response, cell-mediated immune response, and humoral immune response, which involved 90 gene products. This network demonstrated the connectivity of genes in MAPK, NF-κB pathways, as well as the link between MHC class I molecule, complement pathway, cytokines, and chemokines. Molecules are represented as nodes, and the biological relationship between two nodes is represented as an edge (line). Green, down-regulated genes; red, up-regulated genes; gray, not differentially expressed; solid line, direct interaction; dashed line, indirect interaction; yellow line, merged interaction. (TIF) [file pone.0091473.s001.tif]

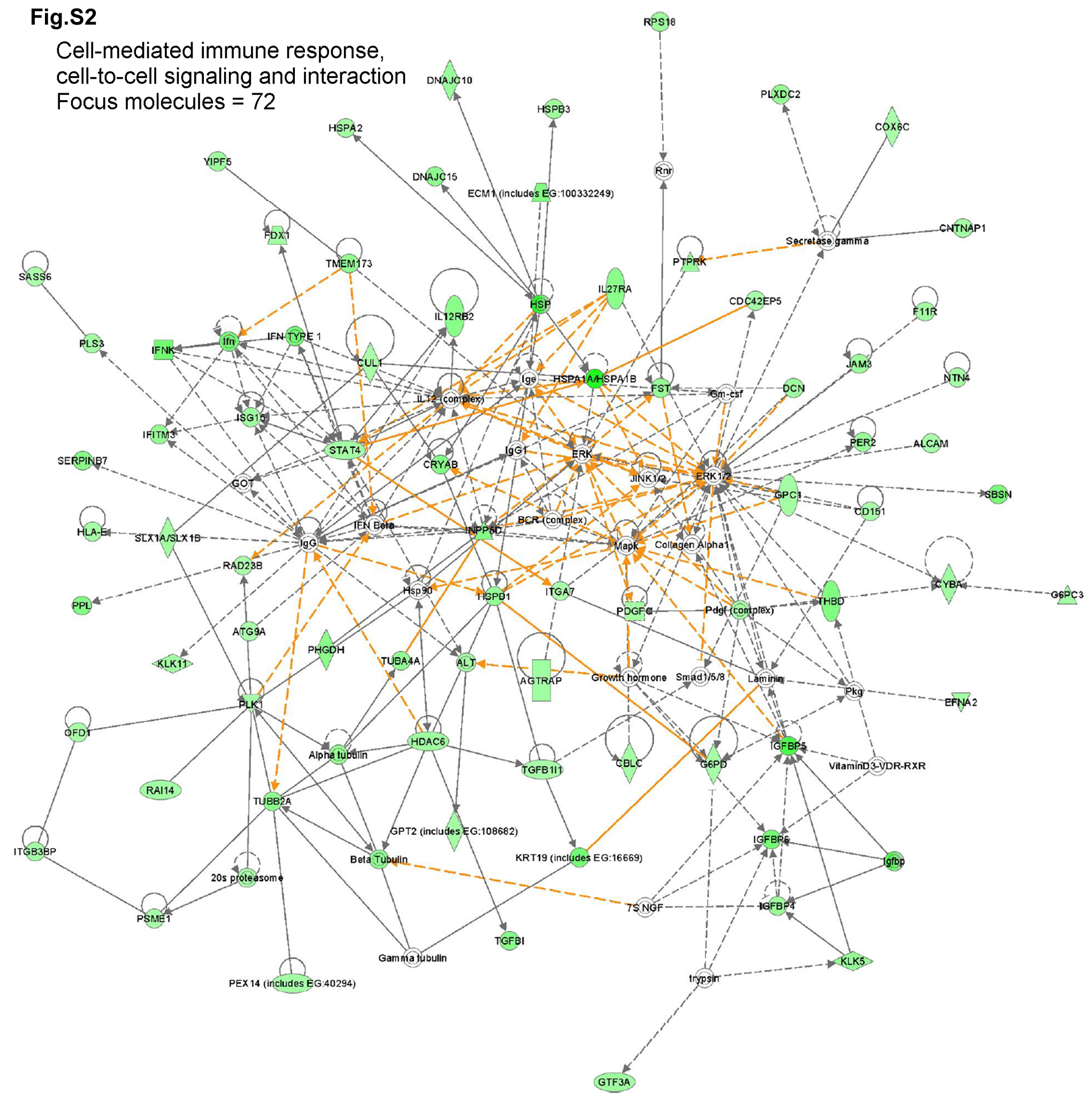

Supplement: Figure S2 — The cell-mediated immune responses-associated network. The network was merged from 72 E2 mediated down-regulated cellular genes based on cell-mediated immune response and cell-to-cell signaling and interaction. This network showed a connection between IFN-κ and STING (TMEM173) with type I IFN being central. Molecules are represented as nodes, and the biological relationship between two nodes is represented as an edge (line). Green, down-regulated genes; gray, not differentially expressed; solid line, direct interaction; dashed line, indirect interaction; yellow line, merged interaction. (TIF) [file pone.0091473.s002.tif]
