# Supplementary material for: E2 Proteins of High Risk Human Papillomaviruses Down-Modulate STING and IFN-κ Transcription in Keratinocytes
Source: PLoS One. 2014 Mar 10;9(3):e91473. doi: 10.1371/journal.pone.0091473 (PMC3948877; doi:10.1371/journal.pone.0091473)
Supplement: Table S1 — Immune responses associated genes modulated by HPV16 E2 in HPK. Gene Ontology analysis indicated that 21 up-regulated genes and 71 down-regulated genes by HPV16 E2 are involved in immune responses. (DOC) [file pone.0091473.s003.doc]

**Table S1. Immune responses associated genes modulated** by HPV16E2 in HPK

| **Accession no.** | **Symbol** | **Name** | **Fold-change** |
| --- | --- | --- | --- |
| NM_004591 | CCL20 | Chemokine (C-C motif) ligand 20 | 10.0 |
| NM_000584 | IL8 | Interleukin 8 | 8.9 |
| NM_003528 | HIST2H2BE | Histone cluster 2, H2be | 4.9 |
| NM_000600 | IL6 | Interleukin 6 (interferon, beta 2) | 4.0 |
| NM_001511 | CXCL1 | Chemokine (C-X-C motif) ligand 1 (melanoma growth stimulating activity, alpha) | 3.8 |
| NM_000594 | TNF | Tumor necrosis factor | 3.0 |
| NM_001172633 | OLR1 | Oxidized low density lipoprotein (lectin-like) receptor 1 | 2.5 |
| NM_006850 | IL24 | Interleukin 24 | 2.5 |
| NM_004233 | CD83 | CD83 molecule, BL11, HB15 | 2.3 |
| NM_012072 | CD93 | CD93 molecule, C1QR1; C1qRP; CDw93; ECSM3; MXRA4; C1qR(P); dJ737E23.1 | 1.9 |
| NM_002994 | CXCL5 | Chemokine (C-X-C motif) ligand 5 | 1.8 |
| NM_000759 | CSF3 | Colony stimulating factor 3 (granulocyte) | 1.8 |
| NM_020529 | NFKBIA | Nuclear factor of kappa light polypeptide gene enhancer in B-cells inhibitor, alpha | 1.7 |
| NM_002993 | CXCL6 | Chemokine (C-X-C motif) ligand 6 (granulocyte chemotactic protein 2) | 1.7 |
| NM_005894 | CD5L | CD5 molecule-like | 1.6 |
| NM_003745 | SOCS1 | Suppressor of cytokine signaling 1 | 1.6 |
| NM_000064 | C3 | Complement component 3 | 1.6 |
| NM_182908 | DHRS2 | Dehydrogenase/reductase (SDR family) member 2 | 1.5 |
| NM_004877 | GMFG | Glia maturation factor, gamma | 1.5 |
| NM_001114182 | IRAK4 | Interleukin-1 receptor-associated kinase 4 | 1.5 |
| NM_012445 | SPON2 | Spondin 2, extracellular matrix protein | 1.5 |
| NM_003019 | SFTPD | Surfactant protein D | -1.5 |
| NM_005052 | RAC3 | Ras-related C3 botulinum toxin substrate 3 (rho family, small GTP binding protein Rac3) | -1.5 |
| NM_145057 | CDC42EP5 | CDC42 effector protein (Rho GTPase binding) 5 | -1.5 |
| NM_001569 | IRAK1 | Interleukin-1 receptor-associated kinase 1 | -1.5 |
| NM_194463 | RNF128 | Ring finger protein 128, E3 ubiquitin protein ligase | -1.5 |
| NM_000402 | G6PD | Glucose-6-phosphate dehydrogenase | -1.6 |
| NM_001127500 | MET | Met proto-oncogene (hepatocyte growth factor receptor) | -1.6 |
| NM_015720 | PODXL2 | Podocalyxin-like 2 | -1.6 |
| NM_004625 | WNT7A | Wingless-type MMTV integration site family, member 7A | -1.6 |
| NM_006263 | PSME1 | Proteasome (prosome, macropain) activator subunit 1 (PA28 alpha) | -1.6 |
| NM_005516 | HLA-E | Major histocompatibility complex, class I, E | -1.6 |
| NM_012116 | CBLC | Cbl proto-oncogene, E3 ubiquitin protein ligase C | -1.6 |
| NM_145160 | MAP2K5 | Mitogen-activated protein kinase kinase 5 | -1.6 |
| NM_003151 | STAT4 | Signal transducer and activator of transcription 4 | -1.6 |
| NM_001139457 | BCAP31 | B-cell receptor-associated protein 31 | -1.6 |
| NM_016946 | F11R | F11 receptor | -1.6 |
| NM_032310 | C9ORF89 | Chromosome 9 open reading frame 89 | -1.6 |
| NM_012290 | TLK1 | Tousled-like kinase 1 | -1.6 |
| NM_000043 | FAS | Fas (TNF receptor superfamily, member 6) | -1.7 |
| NM_000454 | SOD1 | Superoxide dismutase 1, soluble | -1.7 |
| NM_005433 | YES1 | v-yes-1 Yamaguchi sarcoma viral oncogene homolog 1 | -1.7 |
| NM_000101 | CYBA | Cytochrome b-245, alpha polypeptide | -1.7 |
| NM_022372 | MLST8 | MTOR associated protein, LST8 homolog (S. cerevisiae) | -1.7 |
| NM_016818 | ABCG1 | ATP-binding cassette, sub-family G (WHITE), member 1 | -1.7 |
| NM_001271946 | SLIT3 | Slit homolog 3 (Drosophila) | -1.7 |
| NM_005101 | ISG15 | ISG15 ubiquitin-like modifier | -1.7 |
| NM_001098531 | RAPGEF3 | Rap guanine nucleotide exchange factor (GEF) 3 | -1.7 |
| NM_006044 | HDAC6 | Histone deacetylase 6 | -1.7 |
| NM_001199346 | C18ORF32 | Chromosome 18 open reading frame 32 | -1.7 |
| NM_005343 | HRAS | v-Ha-ras Harvey rat sarcoma viral oncogene homolog | -1.7 |
| NM_001552 | IGFBP4 | Insulin-like growth factor binding protein 4 | -1.7 |
| NM_173216 | ST6GAL1 | ST6 beta-galactosamide alpha-2,6-sialyltranferase 1 | -1.8 |
| NM_032801 | JAM3 | Junctional adhesion molecule 3 | -1.8 |
| NM_018981 | DNAJC10 | DnaJ (Hsp40) homolog, subfamily C, member 10 | -1.8 |
| NM_001113490 | AMOT | Angiomotin | -1.8 |
| NM_001012398 | AKTIP | AKT interacting protein | -1.8 |
| NM_006547 | IGF2BP3 | Insulin-like growth factor 2 mRNA binding protein 3 | -1.8 |
| NM_000050 | ASS1 | Argininosuccinate synthase 1 | -1.8 |
| NM_001116 | ADCY9 | Adenylate cyclase 9 | -1.8 |
| NM_014184 | CNIH4 | Cornichon homolog 4, HSPC163 | -1.9 |
| NM_021158 | TRIB3 | Tribbles homolog 3 (Drosophila) | -1.9 |
| NM_001540 | HSPB1 | Heat shock 27kDa protein 1 | -1.9 |
| NM_021034 | IFITM3 | Interferon induced transmembrane protein 3 | -1.9 |
| NM_001779 | CD58 | CD58 molecule, LFA3 | -1.9 |
| NM_002306 | LGALS3 | Lectin, galactoside-binding, soluble, 3 | -1.9 |
| NM_001143688 | DIS3L | DIS3 mitotic control homolog (S. cerevisiae)-like | -1.9 |
| NM_001177676 | GPR68 | G protein-coupled receptor 68 | -2.0 |
| NM_000313 | PROS1 | Protein S (alpha) | -2.0 |
| NM_198282 | TMEM173 | Transmembrane protein 173, ERIS; MITA; MPYS; NET23; STING; FLJ38577 | -2.0 |
| NM_001067 | TOP2A | Topoisomerase (DNA) II alpha 170kDa | -2.0 |
| NM_025217 | ULBP2 | UL16 binding protein 2, N2DL2, RAET1H | -2.0 |
| NM_178812 | MTDH | Metadherin | -2.0 |
| NM_005013 | NUCB2 | Nucleobindin 2 | -2.1 |
| NM_004843 | IL27RA | Interleukin 27 receptor, alpha | -2.1 |
| NM_182663 | RASSF5 | Ras association (RalGDS/AF-6) domain family member 5 | -2.1 |
| NM_001559 | IL12RB2 | Interleukin 12 receptor, beta 2 | -2.1 |
| NM_015973 | GAL | Galanin/GMAP prepropeptide | -2.1 |
| NM_020311 | CXCR7 | Chemokine (C-X-C motif) receptor 7 | -2.1 |
| NM_001017915 | INPP5D | Inositol polyphosphate-5-phosphatase, 145kDa | -2.1 |
| NM_002305 | LGALS1 | Lectin, galactoside-binding, soluble, 1 | -2.1 |
| NM_001001788 | RAET1G | Retinoic acid early transcript 1G | -2.2 |
| NM_000361 | THBD | Thrombomodulin | -2.2 |
| NM_004425 | ECM1 | Extracellular matrix protein 1 | -2.2 |
| NM_005218 | DEFB1 | Defensin, beta 1 | -2.2 |
| NM_015364 | LY96 | Lymphocyte antigen 96 | -2.3 |
| NM_020428 | SLC44A2 | Solute carrier family 44, member 2 | -2.5 |
| NM_020124 | IFNK | Interferon, kappa | -2.5 |
| NM_004864 | GDF15 | Growth differentiation factor 15 | -2.8 |
| NM_000599 | IGFBP5 | Insulin-like growth factor binding protein 5 | -3.2 |
| NM_000090 | COL3A1 | Collagen, type III, alpha 1 | -3.2 |
| NM_003714 | STC2 | Stanniocalcin 2 | -4.0 |
